# Application of γ‐aminobutyric acid (GABA) and nitrogen regulates aroma biochemistry in fragrant rice

**DOI:** 10.1002/fsn3.1240

**Published:** 2019-10-22

**Authors:** Wenjun Xie, Umair Ashraf, Dating Zhong, Rongbin Lin, Peiqi Xian, Tong Zhao, Huoyi Feng, Shuli Wang, Meiyang Duan, Xiangru Tang, Zhaowen Mo

**Affiliations:** ^1^ College of Agriculture South China Agricultural University Guangzhou China; ^2^ Department of Botany Division of Science and Technology University of Education Lahore, Punjab Pakistan; ^3^ Scientific Observing and Experimental Station of Crop Cultivation in South China Ministry of Agriculture, P. R. China Guangzhou China

**Keywords:** 2‐acetyl‐1‐pyrroline, enzyme activity, fragrant rice, nutrient contents, γ‐aminobutyric acid

## Abstract

The 2‐acetyl‐1‐pyrroline (2AP) is a key aroma compound in fragrant rice. The present study assessed the γ‐aminobutyric acid (GABA) and nitrogen (N) application induced regulations in the biochemical basis of rice aroma formation. Four N levels, that is, 0, 0.87, 1.75, and 2.61 g/pot, and two GABA treatments, that is, 0 mg/L (GABA0) and 250 mg/L (GABA250), were applied to three fragrant rice cultivars, that is, Yuxiangyouzhan, Yungengyou 14, and Basmati‐385. Results showed that GABA250 increased 2AP, Na, Mn, Zn, and Fe contents by 8.44%, 10.95%, 25.70%, 11.14%, and 43.30%, respectively, under N treatments across cultivars. The GABA250 further enhanced the activities of proline dehydrogenase (PDH), ornithine aminotransferase (OAT) (both at 15 days after heading (d AH), and diamine oxidase (DAO) (at maturity) by 20.36%, 11.24%, and 17.71%, respectively. Significant interaction between GABA and N for Mn, Zn, and Fe contents in grains, proline content in leaves, GABA content in leaves at 15 d AH and maturity stage (MS), Δ1‐pyrroline‐5‐carboxylic acid (P5C) contents in leaves at 15 d AH, and Δ1‐pyrroline‐5‐carboxylate synthase (P5CS), PDH, and OAT activities in leaves at MS was noted. Moreover, the 2AP contents in grains at MS showed a significant and positive correlation with the proline contents in the leaves at 15d AH. In conclusion, GABA250 enhanced the 2AP, Na, Mn, Zn, and Fe contents, as well as the enzyme activities involved in 2AP biosynthesis. Exogenous GABA and N application improved the 2AP contents and nutrient uptake in fragrant rice.

## 1 INTRODUCTION

Rice is an important cereal crop, which fulfills the dietary needs of billions of people globally (Mahajan, Sekhon, Singh, Kaur, & Sidhu, 2010), whereas fragrant rice is well praised and liked by the consumers due to its high grain quality, strong aroma, and good taste (Chutipaijit & Sutjaritvorakul, 2018; Sasmal & Pal, 2018). “Basmati” and “Jasmine” are the typical fragrant rice types, which are world‐famous due to their long‐grain and fragrant characters. Previous studies have revealed that hundreds of volatile compounds have been detected in aromatic rice, among them 2‐acetyl‐1‐pyrroline (2AP) was recognized as a key aroma compound (Maraval et al., 2008; Shao et al., 2013).

The betaine‐aldehyde dehydrogenase (*BADH*)‐related gene was found to be involved in aroma formation in fragrant rice (Bradbury, Fitzgerald, Henry, Jin, & Waters, 2005; Bradbury, Gillies, Brushett, Waters, & Henry, 2008; Chen et al., 2008; Fitzgerald, Hamilton, Calingacion, Verhoeven, & Butardo, 2008; Kovach, Calingacion, Fitzgerald, & McCouch, 2009; Niu et al., 2008). Previous study revealed the activities of proline dehydrogenase (PDH), Δ1‐pyrroline‐5‐carboxylate synthase (P5CS), ornithine aminotransferase (OAT), and diamine oxidase (DAO), as well as the concentrations of proline and Δ1‐pyrroline‐5‐carboxylic acid (P5C) contents, were involved in 2AP formation in fragrant rice (Ghosh & Roychoudhury, 2018; Huang et al., 2008; Li, Ashraf, et al., 2016; Mo, Fan, et al., 2016; Mo, Huang, et al., 2016; Poonlaphdecha et al., 2012).

The biosynthesis and regulations of 2AP are largely affected by not only the genetic factors but also the external environment and crop management factors (Bao et al., 2018; Hasanuzzaman et al., 2019). The genetic factors play an important part in determining the quality of aromatic rice (Bao et al., 2018; Bradbury et al., 2008), which, on the other hand, are much dependent on prevailing environmental conditions during the growth period and also by pre‐ and postharvest management techniques (Gay et al., 2010). For example, nitrogen fertilization has great impacts on 2AP formation and accumulation. Recently, Ren et al. (2017) showed that 60 kg/hm^2^ nitrogen with water deficit conditions at tillering stage could enhance the 2AP contents in grains significantly, whereas Mo et al. (2018) also demonstrated that different nitrogen application levels affected the 2AP contents in fragrant rice differently. Early studies revealed that BADH induced the biosynthesis of γ‐aminobutyric acid (GABA) by γ‐aminobutyraldehyde, while the inactivation of BADH in aromatic rice induced the accumulation of **△**1‐pyrroline (Bradbury et al., 2008). Moreover, the 2AP and GABA contents in grain were increased under salt treatment and shading condition (Mo et al., 2015; Poonlaphdecha et al., 2012) and there was a significant positive correlation between 2AP and GABA in the grains of “Yuxiangyouzhan” rice cultivar (Mo et al., 2015). Hence, there might be a possibility for exogenous GABA to regulate the 2AP biosynthesis.

GABA is well known as a plant signaling molecule and is involved in various physio‐biochemical processes in plants (Fait, Fromm, Walter, Galili, & Fernie, 2008; Routray & Rayaguru, 2018). GABA has been reported to increase plant resistance ability against several environmental stresses (Yang, Shewfelt, Lee, & Kays, 2008). Regulatory effects of GABA in plant physio‐biochemical metabolism have been previously reported in Arabidopsis, tobacco, maize, barley, and rice (Batushansky et al., 2014; Li, Guo, Yang, Meng, & Wei, 2016; Nayyar, Kaur, Kaur, & Singh, 2014; Song, Xu, Wang, Wang, & Tao, 2010; Yu & Sun, 2007), whereas exogenous GABA applications could improve salt stress resistance in wheat seedlings (Li, Guo, et al., 2016) and enhance photosynthesis capacity in maize seedling (Li, Liu, et al., 2016). In sum, GABA plays a crucial role in plant growth to respond to the external environmental conditions (Fait et al., 2008; Li, Guo, et al., 2016); nevertheless, endogenous GABA levels in plants are very low, but it can be accelerated under stress conditions (Kinnersley & Turano, 2000). Hence, GABA may influence the physiological metabolism of fragrant rice by affecting either aroma biosynthesis or plant growth. Reports are available for GABA‐induced regulations in stress tolerance in various crops; however, effects of exogenous GABA application coupled with various N levels on aroma biosynthesis are rarely investigated. Therefore, the present study was aimed to evaluate the effects of GABA under various levels of N on physio‐biochemical attributes, aroma biosynthesis, and enzyme involved in 2AP formation in fragrant rice.

## 2 MATERIALS AND METHODS

### 2.1 Plant material and growing conditions

The seeds of three aromatic rice cultivars, that is, Yuxiangyouzhan (YX), Yugengyou14 (YG), and Basmati‐385 (BS), were collected from the College of Agriculture, South China Agricultural University, Guangzhou, China. These aromatic rice cultivars are newly developed and famous countrywide due to their aroma. A pot experiment was performed in the greenhouse of the Experimental Research Farm, College of Agriculture, South China Agricultural University, Guangzhou, China, during the early growing season of 2017. Rice seedlings (30 days old) were transplanted into the soil‐containing pots (31cm in diameter and 29 cm in height) on 4 April. The experimental soil was sandy loam containing soil organic matter 18.65 g/kg, total nitrogen 1.17 g/kg, available phosphorus 32.69 mg/kg, available potassium 185.28 mg/kg, and pH 6.44. The yearly average temperature of the region lies between 21 and 29°C and is characterized by a subtropical monsoonal type of climate.

### 2.2 Treatments and plant sampling

The treatment without GABA application was denoted as GABA0 (double‐distilled water sprinkler application), and the GABA at 250mg/L (GABA250) was applied at panicle initiation stage, whereas four nitrogen rates, that is, 0, 0.87, 1.75, and 2.61 g/pot denoted as ZN, LN, MN, and HN, were applied as basal dose. Flag leaves at the heading stage (HS), 15 days after heading stage (15d AH), and maturity stage (MS), whereas mature panicles at MS were collected and stored at ‒80°C for physio‐biochemical assays.

### 2.3 Determination of 2AP contents in grains

The 2AP concentration was determined by synchronization distillation and extraction method (SDE) combined with GCMS‐QP 2010 Plus (Shimadzu Corporation) according to Huang et al. (2012) and the contents of 2AP were expressed as μg/g.

### 2.4 Determination of Na, Mn, Zn, and Fe contents in grains

The Na, Mn, Zn, and Fe contents in grains were determined according to the method of Wu et al. (2014) with some modification. In brief, the grain sample was oven‐dried at 80°C to constant weight and ground in powder, and then weighed 0.25 g of the sample. The weighted samples were dry‐digested in a muffle furnace at 500°C for 6 hr, and then added to 40 ml HNO_3_:H_2_O (1:1). The contents of Na, Mn, Zn, and Fe were determined using a flame atomic absorption spectrometry (SHIMADZU AA‐6300C AA spectrometer).

### 2.5 Measurement of protein contents in grains

After sun drying, about 1.5 kg grains from each treatment were taken to estimate the protein contents by using an Infratec 1241 grain analyzer (FOSS‐TECATOR; Mo et al., 2015).

### 2.6 Determination of proline, GABA, and pyrroline‐5‐carboxylic acid (P5C) contents in leaves

The proline contents in fresh leave samples were determined by Bates, Waldren, and Teare (1973) by using ninhydrin, and the absorbance was read at 520nm. The final proline contents were expressed as μg/g fresh weight (FW) of leaves. The GABA contents were measured according to the methods described by Zhao et al. (2009), and the GABA contents were expressed as μg/g. The P5C concentration was estimated by following the methods of Wu, Chou, Wu, Chen, and Huang (2009). The reaction mixture contained 0.2 ml supernatant of enzyme extract, 0.5 ml of 10% trichloroacetic acid (TCA), and 0.2 ml of 40 mM 2‐aminobenzaldehyde. The absorbance was read at 440 nm, and the contents were expressed as μmol/g.

### 2.7 Determination of the activities of proline dehydrogenase (PDH), △1‐pyrroline‐5‐carboxylic acid synthetase (P5CS), ornithine aminotransferase (OAT), and diamine oxidase (DAO)

The PDH activity was assayed by following the methods of Tateishi, Nakagawa, and Esaka (2005) and Ncube, Finnie, and Van (2013). The absorbance after reaction was read, and the reaction mixture contained L‐proline (15 mM), cytochrome c (0.01 mM), phosphate buffer (100m M, pH 7.4), 0.5% (v/v) Triton X‐100, and the enzyme extract (0.1 ml) in a total volume of 0.5 ml. The reaction mixture was incubated at 37°C for 30 min, and the reaction was terminated by adding 0.5 ml of 10% trichloroacetic acid (TCA). After adding 0.5ml of 0.5% 2‐aminobenzaldehyde in 95% ethanol, the mixture was further incubated at 37°C for 10 min and centrifuged at 8,000 rpm for 10 min, and the absorbance of the supernatant was read at 440 nm, the absorbance change of 0.1 in one minute was defined as one unit of enzyme activity, and the activity was expressed as U/g FW.

The activity of P5CS was estimated according to the methods described by Zhang, Lu, and Verma (1995). The reaction mixture comprised of 50 mM Tris‐HCL buffer, 20 mM MgCl_2_, 50 mM sodium glutamate, 10 mM ATP, 100 mM hydroxamate–HCL, and 0.5 ml of enzyme extract. The reaction was started by the addition of 0.5ml of enzymatic extracts. After 5 min at 37°C, the reaction was stopped by addition of 0.5 ml of a stop buffer (2.5% of FeCl_3_ plus 6% of trichloroacetic acid, dissolved in 100 ml of 2.5 M HCl). The absorbance after the reaction was read at 440 nm, the absorbance change of 0.1 in one minute was defined as one unit of enzyme activity, and the activity was expressed as U/g FW.

The OAT activity was measured according to the methods of Chen, Chen, Lin, and Kao (2001) and Umair, Leung, Bland, and Simpson (2011). The reaction medium contained 100 mM potassium phosphate buffer pH 8.0, 50 mM ornithine, 20 mM α‐ketoglutarate, 1 mM pyridoxal 5‐phosphate, and the enzyme extract (0.1ml)—the final total volume was 1 ml. The reaction medium was incubated at 37°C for 30 min. The reaction was stopped by adding 0.5 ml trichloroacetic acid (10%), and the color was developed by incubating the reaction mixture with 0.5 ml o‐amino benzaldehyde (0.25%) in ethanol (95%) for 1 hr. After centrifugation at 8,000 rpm for 10 min, the clear supernatant fraction was taken to measure the absorbance at 440 nm. The absorbance change of 0.1 in one minute was defined as one unit of enzyme activity; the activity was expressed as U/g FW.

The DAO activity was assayed by using the methods described by Su, An, Zhang, and Liu (2005). The reaction solutions (3.0 ml) contained 2.5 ml 0.1 M sodium phosphate buffer (pH 6.5), 0.1 ml crude enzyme extracts, 0.1 ml peroxidase (250 U/ml), and 0.2 ml 4‐aminoantipyrine/ N, N‐dimethylaniline reaction solutions. The reaction was initiated by the addition of 0.1 ml 20 mM Put. A 0.01 value of the changes in absorbance at 440 nm was regarded as one activity unit of the enzyme, and the activity was expressed as U/g FW.

### 2.8 Statistical analyses

The pots were arranged in a completely randomized design (CRD), and the data were analyzed by using Statistix version 8 (Analytical Software). Relationships among the indexes were evaluated using correlation analyses by Statistix version 8 (Analytical Software). Means among treatments were compared based on the least significant difference test (LSD) at the 0.05 probability level.

## 3 RESULTS

### 3.1 2AP contents

**Figure 1:**
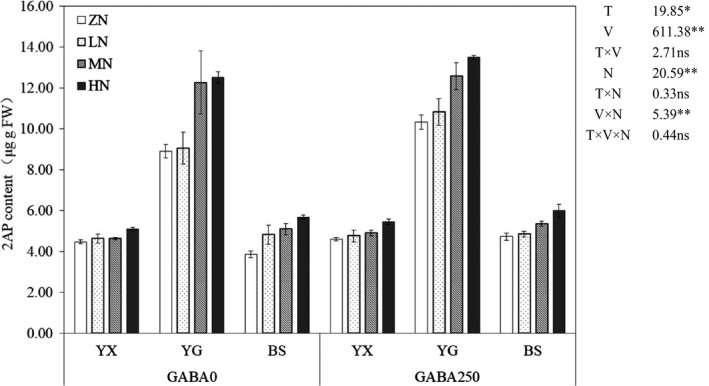
Effect of exogenous γ‐aminobutyric acid (GABA) and nitrogen on 2‐acetyl‐1‐pyrroline contents in grains of three fragrant rice cultivars. T: GABA treatment, V: variety, N: nitrogen levels. Capped bars above means are SE of four replicates

The 2AP contents in grains were substantially affected by GABA treatment (T), variety (V), nitrogen (N), and V × N. Compared with GABA0, the GABA 250 significantly increased the 2AP contents by 8.44% under nitrogen treatments across cultivars. With the increase in the application dose of nitrogen fertilizer, the 2AP contents in grains of all rice cultivars were increased. Moreover, the highest 2AP contents were recorded in YG, which were in the range of 8.89 to 12.57μg/g FW, while the BS and YX were found statistically similar (*p *˃ .05) regarding grain 2AP contents with a range from 3.86 to 5.98 μg/g FW (Figure 1).

### 3.2 Protein contents

**Figure 2:**
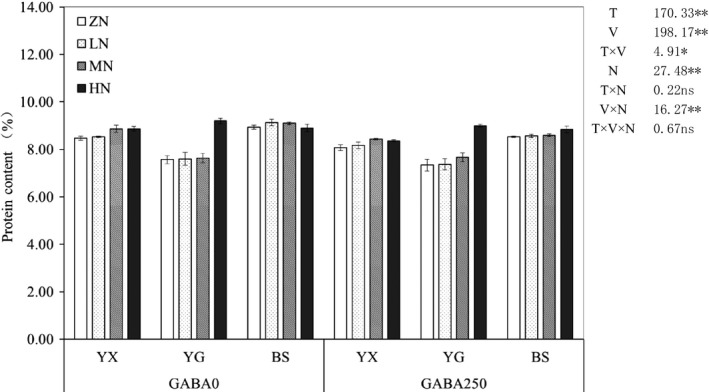
Effect of γ‐aminobutyric acid （GABA） and nitrogen on protein in grains in different fragrant rice genotypes. T represents GABA treatment, V represents variety, and N represents nitrogen treatment. Capped bars above means are SE of four replicates

The T, V, N, T × V, and V × N significantly affected the protein contents in grains of aromatic rice cultivars. Compared with GABA0, GABA250 led to a decrease in the protein content by 3.76% under N treatments across cultivars. With the increased application dose of N fertilizer, the mean protein contents in grains were increased. BS showed higher mean protein content than YX and YG (Figure 2).

### 3.3 Na, Mn, Zn, and Fe contents in grains

Compared with GABA0, Na content in grains under GABA250 was significantly higher, which was 5.33%. Similar trends were also recorded for Mn, Zn, and Fe contents. Compared with GABA0, the Mn, Zn, and Fe contents under nitrogen treatments across cultivars were increased by 25.72%, 11.14%, and 43.30%, respectively, under GABA250. Moreover, GABA and nitrogen interaction affected Na, Mn, Zn, and Fe content in grains significantly. For YX, higher Na, Mn, Zn, and Fe content in grains were found under LN (124.48 mg/kg), ZN (132.33 mg/kg), HN (11.64 mg/kg), and ZN (63.78 mg/kg) under GABA0 treatment, respectively, while higher Na, Mn, Zn, and Fe content in grains were found under HN (188.95 mg/kg), ZN (180.85 mg/kg), ZN (35.89 mg/kg), and ZN (102.38 mg/kg), respectively. For YG, higher Na, Mn, Zn, and Fe content in grains were found under LN, ZN, and ZN, respectively, and HN and MN treatment accumulated highest Zn content under GABA0 and GABA250 treatment, respectively. For BS, highest Mn, Zn, and Fe contents in grains were found under ZN treatment, and HN and LN treatment accumulated highest Zn content under GABA0 and GABA250 treatment, respectively (Table 1).

**Table 1:** Effect of γ‐aminobutyric acid (GABA) and nitrogen on Na, Mn, Zn, and Fe contents in grains in different fragrant rice genotypes

GABA treatment	Cultivars	N dose	Na content	Mn content	Zn content	Fe content
			(mg/kg)			
GABA0	YX	ZN	112.53	132.33	9.64	63.78
		LN	124.48	125.73	9.78	58.27
		MN	105.85	106.60	10.87	42.75
		HN	92.01	104.90	11.64	41.94
	YG	ZN	113.97	237.95	9.49	147.71
		LN	120.76	169.09	9.62	94.21
		MN	107.32	123.59	6.22	58.20
		HN	93.33	130.07	14.14	63.45
	BS	ZN	155.02	149.33	30.27	79.11
		LN	162.96	102.75	22.89	42.34
		MN	140.89	112.28	27.42	47.36
		HN	165.40	88.73	22.23	30.65
		Mean	124.54b	131.95b	15.35b	64.15b
GABA250	YX	ZN	147.25	180.85	35.89	102.38
		LN	177.61	143.66	30.51	72.32
		MN	114.94	86.28	22.21	31.17
		HN	188.95	112.82	24.77	46.98
	YG	ZN	128.14	294.57	10.23	199.15
		LN	134.57	106.76	10.14	48.17
		MN	129.20	139.38	10.27	70.75
		HN	128.44	148.48	9.68	79.33
	BS	ZN	119.27	270.10	20.75	174.17
		LN	147.35	152.56	11.82	79.82
		MN	122.21	176.54	9.62	97.80
		HN	120.20	178.70	8.84	101.05
		Mean	138.18a	165.89a	17.06a	91.93a
ANOVA		T	7.51*	51.78**	4.46	54.00**
		V	10.24*	137.77**	205.45**	108.76**
		T × V	1.61ns	5.44*	1.71ns	7.39**
		*N*	3.*96*	66.51**	9.06**	59.39**
		T × *N*	1.42ns	11.09**	4.68*	11.34**
		V × *N*	1.74ns	2.31ns	3.98*	2.03ns
		T × V × *N*	0.53ns	9.00**	2.68*	8.59**

Note

Means with different lowercase letter(s) differ statistically at *p* < .05.

Abbreviations: BS, Basmati‐385; YG, Yugengyou14; YX, Yuxiangyouzhan.

* Significant at *p* < .05;

** Significant at *p* < .01; ns: nonsignificant.

### 3.4 Proline contents in leaves

Proline content in leaves at HS, 15 d AH, and MS were significantly affected by V, N, T × N, V × N, and T × V×N. The T × V significantly affected the proline content in leaves at HS and 15 d AH. At GABA250, the proline contents in leaves were substantially reduced MS. For YX, under GABA0 treatment, proline content in MN, HN, and HN treatment was the highest at HS, 15 d AH, and MS, respectively, while under GABA250 treatment, proline content in HN, HN, and ZN treatment was the highest at HS, 15 d AH, and MS, respectively. For YG, ZN treatment resulted in the highest proline contents in leaves at HS under GABA0 treatment, while HN treatment produced the highest proline content in leaves at 15 d AH and MS. The proline contents in leaves under HN treatment remained the highest under GABA250 treatment where the proline contents were 14.404, 15.096, and 5.690 µg/g FW for HS, 15 d AH, and MS, respectively. For BS, MN produced the highest proline content in leaves at HS; ZN and HN resulted in the highest proline in leaves at 15 d AH under GABA0 and GABA250, respectively, and HN and ZN had the highest proline in leaves at MS under GABA0 and GABA250, respectively (Table 2).

**Table 2:** Effect of γ‐aminobutyric acid (GABA) and nitrogen on proline accumulation in leaves in different fragrant rice genotypes (μg/g fresh weight (FW))

Treatment	HS	15d AH	MS
GABA0			
YX			
ZN	21.129	6.988	9.056
LN	19.546	7.207	7.522
MN	27.310	6.300	6.571
HN	15.956	8.245	9.444
YG			
ZN	21.341	10.250	4.837
LN	14.544	11.332	5.257
MN	13.959	13.342	5.109
HN	9.199	14.543	7.708
BS			
ZN	12.915	9.331	7.691
LN	13.901	8.496	8.685
MN	16.443	6.810	8.622
HN	16.300	8.253	11.624
Mean	16.879	9.258	7.677a
GABA250			
YX			
ZN	22.918	8.671	8.357
LN	17.828	6.249	7.058
MN	20.753	8.247	6.814
HN	23.858	9.287	7.796
YG			
ZN	10.180	6.516	4.155
LN	11.692	9.819	4.205
MN	11.196	10.659	5.020
HN	14.404	15.096	5.690
BS			
ZN	13.693	8.280	8.730
LN	14.659	7.866	6.193
MN	16.506	9.113	7.792
HN	16.049	9.662	8.148
Mean	16.145	9.122	6.663b
ANOVA			
T	1.29ns	0.27ns	48.11**
V	76.52**	122.05**	238.41**
T × V	3.94*	17.10**	3.36ns
*N*	5.61**	19.44**	43.24**
T × *N*	15.14**	4.09*	16.37**
V × *N*	6.42**	10.53**	7.69**
T × V × *N*	9.88**	2.66*	4.28**

Note

Means with different lowercase letter(s) differ statistically at *p* < .05.

Abbreviations: BS, Basmati‐385; d AH, days after heading; HS, heading stage; MS, maturity stage; YG, Yugengyou14; YX, Yuxiangyouzhan.

* Significant at *p* < .05;

** Significant at *p* < .01; ns: nonsignificant.

### 3.5 P5C concentration

GABA treatment (T) did not affect the P5C contents in leaves, whereas the cultivars were found statistically significant regarding P5C contents in leaves. Significant differences were observed for T × V, N, T × N, V × N, and T × V×N at 15 d AH. Furthermore, YG produced lower P5C content in leaves as compared to YX and BS, while P5C contents substantially differed among different growth stages (Table 3).

**Table 3:** Effect of γ‐aminobutyric acid (GABA) and nitrogen on P5C accumulation in leaves in different fragrant rice genotypes (µmol/g FW)

Treatment	HS	15d AH	MS
GABA0			
YX			
ZN	0.509	1.085	1.010
LN	0.428	0.857	1.243
MN	0.642	1.015	0.979
HN	0.614	1.355	1.265
YG			
ZN	0.391	0.945	0.534
LN	0.484	0.636	0.511
MN	0.378	0.648	0.639
HN	0.352	0.289	0.367
BS			
ZN	1.228	0.819	0.864
LN	2.148	0.709	1.194
MN	0.673	0.774	0.994
HN	0.657	0.628	0.910
Mean	0.709	0.813	0.876
GABA250			
YX			
ZN	0.545	0.895	0.907
LN	0.494	1.274	0.921
MN	0.426	1.395	1.090
HN	0.548	1.180	1.296
YG			
ZN	0.722	0.488	0.465
LN	0.437	0.547	0.832
MN	0.414	0.560	0.461
HN	0.487	0.456	0.628
BS			
ZN	0.936	1.005	0.746
LN	0.628	0.860	0.781
MN	0.764	1.328	1.130
HN	1.313	0.714	0.815
Mean	0.643	0.892	0.839
ANOVA			
T	0.56ns	6.65ns	0.65ns
V	12.89**	81.69**	31.39**
T × V	1.15ns	8.47**	1.1ns
*N*	0.96ns	3.55*	2.23ns
T × *N*	2.60ns	4.98**	1.02ns
V × *N*	1.01ns	4.32**	1.78ns
T × V × *N*	2.67*	2.88*	2.19ns

Note

Means with different lowercase letter(s) differ statistically at *p* < .05.

Abbreviations: BS, Basmati‐385; d AH, days after heading; HS, heading stage; MS, maturity stage; YG, Yugengyou14; YX, Yuxiangyouzhan.

* Significant at *p* < .05;

** Significant at *p* < .01; ns: nonsignificant.

### 3.6 GABA contents

Cultivar significantly differed regarding the accumulation of GABA contents in leaves; however, GABA treatment (T), nitrogen (N), T × V, V × N, and T × V×N did not affect GABA content in leaves significantly. On the other hand, significant differences were observed in GABA content for T × N at 15 d AH and MS, whereas YG produced lower GABA content in leaves as compared to YX and BS, while GABA contents substantially differed (*p* < .05) among different growth stages (Table 4).

**Table 4:** Effect of γ‐aminobutyric acid (GABA) and nitrogen on GABA content in leaves in different fragrant rice genotypes (µg/g FW)

Treatment	HS	15dAH	MS
GABA0			
YX			
ZN	107.71	86.64	113.94
LN	128.58	94.32	91.73
MN	107.98	94.29	84.59
HN	119.58	91.01	116.99
YG			
ZN	23.34	11.17	7.21
LN	30.99	21.23	7.26
MN	32.81	27.22	7.22
HN	43.60	19.38	9.19
BS			
ZN	112.70	94.30	88.34
LN	118.78	116.35	106.45
MN	110.16	129.76	79.58
HN	110.25	101.03	107.46
Mean	87.21	73.89	68.33
GABA250			
YX			
ZN	126.06	86.50	93.09
LN	107.54	81.45	95.78
MN	135.41	84.23	85.73
HN	120.22	101.48	92.92
YG			
ZN	23.20	23.28	11.37
LN	25.42	13.32	11.13
MN	26.92	23.46	7.13
HN	37.92	15.46	9.11
BS			
ZN	111.75	123.46	136.96
LN	111.03	106.26	113.61
MN	109.41	118.07	106.13
HN	99.29	110.26	80.84
Mean	86.18	73.93	70.32
Analysis of variance			
T	0.22ns	0.91ns	0.18ns
V	745.94**	127.65**	274.74**
T × V	2.87ns	0.15ns	2.15ns
*N*	0.27ns	0.87ns	2.23ns
T × *N*	0.92ns	3.45*	2.92*
V × *N*	1.61ns	0.50ns	1.18ns
T × V × *N*	0.86ns	1.50ns	2.19ns

Note

Means with different lowercase letter(s) differ statistically at *p* < .05.

Abbreviations: BS, Basmati‐385; d AH: days after heading; HS, heading stage; MS: maturity stage; YG: Yugengyou14, YX, Yuxiangyouzhan,

* Significant at *p* < .05;

** Significant at *p* < .01; ns: non‐significant.

### 3.7 Activities of P5CS, PDH, OAT, and DAO

No significant difference was found regarding P5CS activity in leaves under GABA treatment (T). Variety (V) and nitrogen (N) substantially affected the P5CS activity in leaves of all rice cultivars. Significant effect of T × V on P5CS activity in leaves at HS and 15 d AH was observed. Significant interactive effect of V × N and T × V×N on P5CS activity in leaves at 15 d AH and MS and T × N at MS was noted. Lower P5CS activity in leaves was investigated in YG as compared to YX and BS. Under GABA250 treatment, lower nitrogen application improved P5CS activities in the leaves of all aromatic rice cultivars (Table 5).

**Table 5:** Effect of γ‐aminobutyric acid (GABA) and nitrogen on P5CS activity in leaves in different fragrant rice genotypes (U/g FW)

Treatment	HS	15d AH	MS
GABA0			
YX			
ZN	9.859	10.585	14.537
LN	10.166	10.493	13.369
MN	7.455	14.659	14.055
HN	7.670	9.901	10.987
YG			
ZN	6.103	6.362	7.148
LN	6.840	6.054	6.953
MN	5.151	4.491	7.628
HN	6.556	6.712	6.137
BS			
ZN	13.310	48.553	13.517
LN	11.574	17.559	73.011
MN	8.694	13.973	24.057
HN	10.267	56.993	12.069
Mean	8.637	17.195	16.956
GABA250			
YX			
ZN	13.503	12.667	13.251
LN	10.858	11.839	15.622
MN	8.571	10.150	10.333
HN	8.042	12.555	10.082
YG			
ZN	7.653	6.283	8.476
LN	7.171	5.535	8.231
MN	4.673	5.481	6.989
HN	5.444	6.312	7.880
BS			
ZN	11.633	16.082	27.692
LN	11.492	13.046	18.846
MN	9.086	11.955	21.812
HN	7.548	10.929	21.335
Mean	8.806	10.236	14.212
ANOVA			
T	0.12ns	4.10ns	1.90ns
V	53.54**	9.06**	37.81**
T × V	4.13*	4.24*	2.29ns
*N*	17.75**	3.64*	16.09**
T × *N*	1.79ns	2.68ns	16.42**
V × *N*	1.17ns	3.33**	11.76**
T × V × *N*	0.89ns	3.65**	19.55**

Note

Means with different lowercase letter(s) differ statistically at *p* < .05.

Abbreviations: BS, Basmati‐385; d AH, days after heading; HS, heading stage; MS: maturity stage; YG, Yugengyou14; YX, Yuxiangyouzhan.

* Significant at *p* < .05;

** Significant at *p* < .01; ns: nonsignificant.

GABA treatment (T) showed a significant difference in PDH activity in leaves at 15d AH. Variety (V) and T × V significantly affected PDH activity in leaves at HS and 15 d AH. Significant effect of nitrogen (N) on PDH activity in leaves at HS and MS was observed. Remarkable T × N and T × V×N effect on PDH activity in leaves at MS and HS was detected, respectively. Higher PDH activity in leaves was observed under GABA250, and a significant increase in PDH activity in leaves at 15 d AH by 20.364% under GABA250 compared to GABA0 was investigated. Under GABA250 treatment, higher PDH activity in leaves at MS was found for lower N application treatment (Table 6).

**Table 6:** Effect of γ‐aminobutyric acid (GABA) and nitrogen on PDH activity in leaves in different fragrant rice genotypes (U/g FW)

Treatment	HS	15d AH	MS
GABA0			
YX			
ZN	10.199	11.685	8.765
LN	11.310	9.203	8.204
MN	9.132	12.794	12.783
HN	8.364	14.100	16.545
YG			
ZN	12.046	8.838	8.450
LN	10.854	9.262	7.812
MN	9.890	8.341	9.441
HN	7.841	4.548	8.603
BS			
ZN	12.216	12.417	8.933
LN	18.202	12.989	7.712
MN	11.141	15.156	9.650
HN	16.070	11.131	8.168
Mean	11.278	10.872 b	9.589
GABA250			
YX			
ZN	14.581	11.497	13.909
LN	13.485	14.091	17.909
MN	11.184	12.768	9.088
HN	13.766	12.085	8.281
YG			
ZN	8.140	10.907	13.768
LN	10.978	8.476	11.693
MN	10.439	9.232	11.152
HN	11.815	9.101	7.455
BS			
ZN	15.938	18.453	17.408
LN	12.453	15.353	22.175
MN	13.292	16.744	9.976
HN	13.742	18.318	9.430
Mean	12.484	13.086 a	12.687
ANOVA			
T	4.92ns	32.40*	6.39ns
V	52.90**	55.07**	1.89ns
T × V	15.72**	4.56*	2.64ns
*N*	3.06*	0.63ns	3.09*
T × *N*	2.47ns	0.68ns	14.36**
V × *N*	0.84ns	0.89ns	1.69ns
T × V × *N*	4.43**	1.92ns	1.66ns

Note

Means with different lowercase letter(s) differ statistically at *p* < .05.

Abbreviations: BS, Basmati‐385; d AH, days after heading; HS, heading stage; MS: maturity stage; YG, Yugengyou14; YX, Yuxiangyouzhan.

* Significant at *p* < .05;

** Significant at *p* < .01; ns: nonsignificant.

Significant effects of GABA treatment (T) were noted on OAT activity in the leaves of aromatic rice cultivars at 15d AH. Varieties (V) were also differed statistically (*p* < .05) regarding OAT activity, while nitrogen (N) did not affect OAT activity in leaves significantly. Moreover, significant effects of T × V, T × N, and T × V×N on OAT activity in leaves at MS were observed, whereas the T × N effect on OAT activity in leaves at 15 d AH and MS has also remained significant. The OAT activity in the leaves at 15 d AH was increased by 11.242% under GABA250 than GABA0. Under GABA250 treatment, higher OAT activity in leaves was found in ZN for YG and LN and MN for YX (Table 7).

**Table 7:** Effect of γ‐aminobutyric acid （GABA） and nitrogen on OAT activity in leaves in different fragrant rice genotypes (U/g FW)

Treatment	HS	15d AH	MS
GABA0			
YX			
ZN	50.436	118.519	175.335
LN	53.765	131.904	180.132
MN	39.305	167.937	173.495
HN	58.381	154.551	174.264
YG			
ZN	80.354	77.057	88.004
LN	77.795	85.609	98.689
MN	72.791	76.401	176.657
HN	68.798	65.836	187.467
BS			
ZN	137.599	154.256	196.877
LN	157.291	155.757	178.268
MN	135.698	177.890	193.295
HN	143.348	135.035	174.143
Mean	89.630	125.063 b	166.385
GABA250			
YX			
ZN	49.478	142.124	174.679
LN	56.952	162.462	203.454
MN	57.504	182.127	182.437
HN	49.556	157.765	175.051
YG			
ZN	93.848	121.396	112.825
LN	85.471	77.531	108.065
MN	85.621	76.574	93.593
HN	87.751	78.630	90.222
BS			
ZN	154.083	160.150	196.942
LN	148.063	163.815	227.015
MN	156.887	171.943	209.212
HN	134.361	174.965	197.217
Mean	96.631	139.123 a	164.226
ANOVA			
T	6.01ns	12.40*	0.16ns
V	376.67**	140.20**	59.29**
T × V	1.23ns	0.21ns	8.40**
*N*	1.26ns	1.92ns	1.82ns
T × *N*	2.62ns	0.97ns	7.90**
V × *N*	0.89ns	3.05*	3.93**
T × V × *N*	1.39ns	1.38ns	6.02**

Note

Means with different lowercase letter(s) differ statistically at *p* < .05.

Abbreviations: BS, Basmati‐385; d AH, days after heading; HS, heading stage; MS, maturity stage; YG, Yugengyou14; YX, Yuxiangyouzhan.

* Significant at *p* < .05;

** Significant at *p* < .01; ns: nonsignificant.

GABA treatment (T) significantly affected DAO activity in leaves at MS, whereas variety (V) showed differential responses regarding DAO activity. Nitrogen (N) significantly affected DAO activity in all rice cultivars at HS and MS. The interactive effects of V × N and T × V×N on DAO activity in leaves at MS and 15 d AH were found significant, respectively. The DAO activity in the leaves at MS was increased by 17.712% under GABA250 compared to GABA0 (Table 8).

**Table 8:** Effect of γ‐aminobutyric acid (GABA) and nitrogen on DAO activity in leaves in different fragrant rice genotypes (U/g FW)

Treatment	HS	15d AH	MS
GABA0			
YX			
ZN	5.393	5.457	6.350
LN	5.499	5.651	6.929
MN	3.518	6.613	7.199
HN	4.352	5.682	6.114
YG			
ZN	3.569	2.761	5.394
LN	3.232	3.772	5.020
MN	3.518	3.186	4.227
HN	3.069	2.772	3.752
BS			
ZN	2.843	4.553	3.985
LN	4.376	4.386	5.866
MN	3.169	4.062	5.775
HN	2.555	2.981	5.039
Mean	3.758	4.323	5.471 b
GABA250			
YX			
ZN	4.435	5.766	6.158
LN	3.968	7.288	9.200
MN	3.733	6.146	8.288
HN	3.623	6.238	7.803
YG			
ZN	3.897	5.059	6.453
LN	3.858	3.656	5.150
MN	2.446	4.375	5.009
HN	3.491	3.039	4.780
BS			
ZN	3.996	4.622	5.376
LN	3.508	4.661	7.609
MN	4.147	4.777	6.379
HN	2.877	5.525	5.119
Mean	3.665	5.096	6.444 a
ANOVA			
T	0.24ns	9.78ns	15.95*
V	8.59**	46.90**	16.82**
T × V	2.74ns	0.38ns	0.17ns
*N*	4.92**	1.32ns	4.23**
T × *N*	0.93ns	0.49ns	0.29ns
V × *N*	1.58ns	0.81ns	2.86*
T × V × *N*	2.17ns	2.80*	0.99ns

Note

Means with different lowercase letter(s) differ statistically at *p* < .05.

Abbreviations: BS, Basmati‐385; d AH, days after heading; HS, heading stage; MS, maturity stage; YG, Yugengyou14; YX, Yuxiangyouzhan.

* Significant at *p* < .05;

** Significant at *p* < .01; ns: nonsignificant.

### 3.8 Correlation analyses

The associations of micronutrients, that is, Na, Mn, Fe, and protein contents in grains, have remained nonsignificant (P˃0.05) with grain 2AP contents. The grain 2AP contents showed a significant and positive correlation with proline contents in leaves at 15d AH under both GABA0 and GABA250. Moreover, under GABA0, significant negative correlations were found between grain 2AP and P5C contents at 15d AH and MS, PDH activity at 15d AH, OAT activity at 15 d AH, P5CS activity at HS, DAO activity at 15 d AH, DAO activity at MS, GABA content at HS, GABA content at 15 d AH, and GABA content at MS. Under GABA250, significant negative correlation relationship exists between 2AP content in grains and proline content at HS and MS, P5C content at 15d AH and MS, PDH activity at HS and 15d AH, OAT activity at 15 d AH and MS, P5CS activity at HS, 15 d AH, and MS, DAO activity at 15 d AH and MS, and GABA content at HS, 15 d AH, and MS (Table 9).

**Table 9:** Correlation analyses between 2AP content in grains and the investigated indices under GABA0 and GABA250

Investigated parameters	*r*	
	GABA0	GABA250
Na content in grains	−.4746ns	−.2631ns
Mn content in grains	.3313ns	−.0151ns
Zn content in grains	−.4603ns	−.5893*
Fe content in grains	.3417ns	.0134ns
Protein content in grains	−.4748ns	−.3527ns
Proline content at HS	−.4481ns	−.6470*
Proline content at15d AH	.9274**	.6353*
Proline content at MS	−.5366ns	−.7836**
P5C content at HS	−.4523ns	−.3187ns
P5C content at15d AH	−.6096*	−.8059**
P5C content at MS	−.8328**	−.6759*
GABA content at HS	−.8910**	−.9388**
GABA content at 15 d AH	−.8627**	−.9112**
GABA content at MS	−.8924**	−.9406**
PDH activity at HS	−.3483ns	−.6681*
PDH activity at 15d AH	−.8127**	−.7318**
PDH activity at MS	−.2231ns	−.3915ns
OAT activity at HS	−.2374ns	−.1241ns
OAT activity at 15 d AH	−.8752**	−.9272**
OAT activity at MS	−.3232ns	−.9449**
P5CS activity at HS	−.7635**	−.8134**
P5CS activity at 15 d AH	−.4447ns	−.9008**
P5CS activity at MS	−.3725ns	−.6652*
DAO activity at HS	−.3407ns	−.4584ns
DAO activity at 15 d AH	−.7044*	−.7094**
DAO activity at MS	−.6377*	−.6116*

Abbreviation: ns, non‐significant.

*Significant at *p *< .05; **Significant at *p *< .01.

## 4 DISCUSSION

Influences of the GABA and N application regulations in the 2AP and the activities of the enzymes involved in its biosynthesis were assessed in this study. The 2AP is recognized as the key compound for the fragrance of aromatic rice in many previous reports (Buttery, Ling, Juliano, & Turnbaugh, 1983; Magnus, Juliano, & Peter, 2002; Poonlaphdecha et al., 2012). In this study, the 2AP content in grain increased with the improvement of N level and the 2AP content was significantly increased under GABA250 treatment (Figure 1). Previous studies have indicated that salt and shading treatment increased the 2AP and GABA contents in grain (Mo et al., 2015; Poonlaphdecha et al., 2012) and a significant positive correlation between 2AP and GABA in the grains was also observed for the “Yuxiangyouzhan” (Mo et al., 2015). Additionally, nitrogen fertilization has great impacts on 2AP formation and accumulation (Mo et al., 2018; Ren et al., 2017). Therefore, the main reasons that GABA and N regulate the aroma formation are as follows: (a) GABA is directly related to 2AP formation; and (b) the GABA and N regulate the physio‐chemical parameters of 2AP formation.

Moreover, compared with GABA0, Na, Mn, Zn, and Fe contents in grains under GABA250 were substantially increased. This was supported by the study of Kinnersley and Lin (2000), who have reported that the Lemna minor plants treated with 10 mM GABA yielded higher levels of Mn and Zn than the untreated plants. The increase in the content of micronutrients was mainly caused by the enhancement of assimilation capacity, which was induced by GABA application. The interaction of GABA and N affected Na, Mn, Zn, and Fe contents in grains significantly (Table 1).

Proline contents in leaves at HS, 15 d AH, and MS were significantly affected by V, N, T × N, and V × N; however,, the proline contents in leaves under GABA250 treatment were substantially reduced at MS (Table 2), which suggested the transportation of proline from leaves to grains during grain filling and lead to higher 2AP accumulation in grain. So, correlation analysis revealed that the 2AP content in fragrant rice was significantly and positively associated with proline content in leaves at 15 d AH (Table 9). Our results confirm the previous reports in which proline is reported as the precursor of 2AP in fragrant rice, and its higher level often results in more 2AP contents (Huang et al., 2008; Poonlaphdecha et al., 2012). Generally, genetic factors largely determine the aroma formation in fragrant rice (Bradbury et al., 2008; Fitzgerald et al., 2008); nevertheless, many environmental factors and the cultivation practices may have a significant influence on aroma volatiles in rice (Li, Ashraf, et al., 2016; Mo et al., 2015; Yang et al., 2012). Nitrogen fertilizer is also one of the important factors that could lead to the improvement of 2AP accumulation significantly (Ren et al., 2017; Sikdar, Rahman, Islam, Yeasmin, & Akhter, 2008), even there are still different arguments on whether or not N could improve the 2AP content in fragrant rice (Itani, Tamaki, Hayata, Fushimi, & Hashizume, 2004; Li et al., 2014; Yoshihashi, 2005). Moreover, GABA and nitrogen application did not affect the GABA contents in leaves significantly (Table 4), whereas significant but negative correlations between 2AP content in grain and GABA in leaves were detected (Table 9). Differences in opines exist among scientists regarding relationships of GABA with 2AP. For example, Poonlaphdecha et al. (2012) revealed that 2AP was correlated with proline content but not with the GABA content in leaves, whereas the positive correlation between 2AP and GABA contents in fragrant rice grains was detected by Mo et al. (2015). Furthermore, GABA treatment did not affect the P5C contents, whereas the cultivars were found statistically significant regarding P5C contents in leaves of all rice cultivars. Significant differences in P5C contents were observed for T × V, N, T × N, V × N, and T × V×N at 15 d AH (Table 3), whereas GABA treatment enhanced the activities of PDH, OAT at 15 d AH, and DAO at maturity. The significant interaction between GABA and N for P5CS, PDH, and OAT activities in leaves at MS was observed. In addition, GABA treatment enhanced the activities of PDH, OAT at 15 d AH, and DAO at maturity. The significant interaction between GABA and N for P5CS, PDH, and OAT activities in leaves at MS was observed (Tables 5, 6, 7, 8). Moreover, the 2AP contents in grains at maturity showed significant negative associations with some of the investigated enzyme activity in leaves at some growth stage (Table 9). The difference between this study and other previous study is mainly due to the difference in cultivars, application treatments, the correlation analysis that was between 2AP, and the biochemistry parameters in different plant parts. Previous studies reported that grain 2AP contents in fragrant rice are directly associated with the activities of PDH, P5CS, OAT, and DAO (Li, Ashraf, et al., 2016), whereas Ghosh and Roychoudhury (2018) reported that aromatic rice types have higher activities of PDH, P5CS, and OAT and P5C contents than nonaromatic rice types. Furthermore, Mo et al. (2017) also demonstrated that PDH activity and proline content were connected to the 2AP formation and accumulation. Differences in the concentration of 2AP in different plant parts showed their differential abilities to accumulate the 2AP contents (Buttery et al., 1983; Maraval et al., 2010). Micronutrients such as Mn, Zn, and Fe also play important roles in modulation of 2AP contents (Hu, Xu, & Huang, 2001; Huang et al., 2008; Huang, Xiao, & Tang, 2010; Li, Ashraf, et al., 2016; Tang & Wu, 2006). Present study indicated that exogenous GABA application improved the uptake of Na, Mn, Zn, and Fe in all rice cultivars. Overall, GABA application in interaction with N substantially modulated the 2AP contents in grains by affecting the enzyme activities involved in the 2AP formation; however, further studies are needed to better understand the involvement of and/or mechanism of GABA and N to regulate the 2AP biosynthesis and the enzymes involved in the whole process.

## 5 CONCLUSION

GABA250 can increase 2AP, Na, Mn, Zn, and Fe contents, but decrease in protein content in grains as compared to GABA0. The GABA250 treatment further enhanced the activities of PDH and OAT at 15 d AH and DAO activity at maturity but reduced the proline contents at maturity. Significant interactive effect of GABA and nitrogen was observed for Mn, Zn, and Fe content in grains, proline content in leaves, P5C content in leaves at 15 d AH, GABA content in leaves at 15 d AH and MS, and P5CS, PDH, and OAT activities in leaves at MS. Overall, GABA treatment improved the 2AP content and nutrient uptake in all rice cultivars, whereas GABA and nitrogen revealed significant interaction effect on nutrient content in grains and some physiological parameters in leaves that involved in 2AP formation.

## CONFLICT OF INTEREST

The authors declare that they have no conflict of interest.

## ETHICAL APPROVAL

The study did not involve any human or animal testing.

## INFORMED CONSENT

Written informed consent was obtained from all study participants.
